# A Cross-Sectional Study of the Microeconomic Impact of Cardiovascular Disease Hospitalization in Four Low- and Middle-Income Countries

**DOI:** 10.1371/journal.pone.0020821

**Published:** 2011-06-14

**Authors:** Mark D. Huffman, Krishna D. Rao, Andres Pichon-Riviere, Dong Zhao, S. Harikrishnan, Kaushik Ramaiya, V. S. Ajay, Shifalika Goenka, Juan I. Calcagno, Joaquín E. Caporale, Shaoli Niu, Yan Li, Jing Liu, K. R. Thankappan, Meena Daivadanam, Jan van Esch, Adrianna Murphy, Andrew E. Moran, Thomas A. Gaziano, Marc Suhrcke, K. Srinath Reddy, Stephen Leeder, Dorairaj Prabhakaran

**Affiliations:** 1 Departments of Preventive Medicine and Medicine, Northwestern University Feinberg School of Medicine, Chicago, Illinois, United States of America; 2 Centre for Chronic Disease Control, New Delhi, India; 3 Public Health Foundation of India, New Delhi, India; 4 Institute for Clinical Effectiveness Research and Health Policy, Buenos Aires, Argentina; 5 Initiative for Cardiovascular Health Research in Developing Countries, New Delhi, India; 6 Department of Epidemiology, Capital Medical University affiliated Beijing Anzhen Hospital and Beijing Institute of Heart, Lung and Blood Vessel Diseases, Beijing, China; 7 Achutha Menon Centre for Health Science Studies, Sree Chitra Tirunal Institute for Medical Sciences and Technology, Trivandrum, India; 8 Department of Internal Medicine, Shree Hindu Mandal Hospital, Muhimbili University of Health and Allied Sciences, Dar es Salaam, Tanzania; 9 Cadre Ward, Navy General Hospital, Beijing, China; 10 Department of Global Health and Population, Harvard School of Public Health, Boston, Massachusetts, United States of America; 11 Division of General Medicine, Columbia University Medical Center, New York, New York, United States of America; 12 Division of Cardiovascular Medicine, Brigham and Women's Hospital, Boston, Massachusetts, United States of America; 13 School of Medicine, Health Policy and Practice, University of East Anglia, Norwich, United Kingdom; 14 Menzies Centre for Health Policy, University of Sydney, Sydney, Australia; Universidad Peruana Cayetano Heredia, Peru

## Abstract

**Objective:**

To estimate individual and household economic impact of cardiovascular disease (CVD) in selected low- and middle-income countries (LMIC).

**Background:**

Empirical evidence on the microeconomic consequences of CVD in LMIC is scarce.

**Methods and Findings:**

We surveyed 1,657 recently hospitalized CVD patients (66% male; mean age 55.8 years) from Argentina, China, India, and Tanzania to evaluate the microeconomic and functional/productivity impact of CVD hospitalization. Respondents were stratified into three income groups. Median out-of-pocket expenditures for CVD treatment over 15 month follow-up ranged from 354 international dollars (2007 INT$, Tanzania, low-income) to INT$2,917 (India, high-income). Catastrophic health spending (CHS) was present in >50% of respondents in China, India, and Tanzania. Distress financing (DF) and lost income were more common in low-income respondents. After adjustment, lack of health insurance was associated with CHS in Argentina (OR 4.73 [2.56, 8.76], India (OR 3.93 [2.23, 6.90], and Tanzania (OR 3.68 [1.86, 7.26] with a marginal association in China (OR 2.05 [0.82, 5.11]). These economic effects were accompanied by substantial decreases in individual functional health and productivity.

**Conclusions:**

Individuals in selected LMIC bear significant financial burdens following CVD hospitalization, yet with substantial variation across and within countries. Lack of insurance may drive much of the financial stress of CVD in LMIC patients and their families.

## Introduction

Cardiovascular disease (CVD) is the leading cause of mortality and among the leading causes of morbidity worldwide [1]. On average, CVD affects patients in low- and middle-income countries (LMIC) ten to fifteen years earlier than high-income country patients, reducing LMIC workforce capacity and potential economic growth [2]. The number of potentially productive years of life lost (PPYLL) due to CVD between ages 35–64 was estimated to be 9.2 million (3,572 per 100,000) in India and 6.7 million (1,595 per 100,000) in China in 2000. These estimates are projected to rise to 17.9 million PPYLL in India (3,707 PPYLL per 100,000) and 10.5 million PPYLL in China (1,863 per 100,000), by 2030 [2]. On a national scale, Tanzania, India and China are projected to lose 2.5 billion, 237 billion, and 558 billion 1998 international dollars in gross domestic product (GDP), respectively, between 2005–2015 due to CVD, diabetes, and stroke [3]. To the best of our knowledge, there are no similar estimates since 2000.

Beyond these macroeconomic projections, the 2000 World Health Report used fairness in financing—defined as the ratio of a household's total health spending to its capacity to pay—as a key indicator of health system performance (4). In this context, Xu and colleagues estimated in 2007 that 150 million people suffer from financial catastrophe (defined as annual health spending ≥40% non-food income) due to out-of pocket spending on health care. More than 90% of these people live in low-income countries [4]. Despite the high prevalence of CVD and out-of-pocket healthcare financing in LMIC, there are limited data evaluating the association between acute CVD events and their economic impact.

We conducted a standardized survey in four LMICs—Argentina, China, India, and Tanzania—in order to quantify the effect of hospitalization for a CVD event on (a) health care expenditures, (b) how people financed health expenditures (c) impoverishment, and (d) changes in functional capacity and productivity. We hypothesized that a CVD event in a family member would produce a higher effect on family financial stress in low income families and families without health insurance.

## Methods

### Ethics Statement

The study was approved by a local ethics board for each participating site. Specifically, approval was provided by ethics boards located at the following institutions: Sree Chitra Tirunal Institute for Medical Sciences and Technology, National Institute of Medical Research, Muhimbili National Hospital, Capital Medical University affiliated Beijing Anzhen Hospital & Beijing Institute of Heart, Lung and Blood Vessel Diseases, and Hospital Italiano de Buenos Aires. Written informed consent was obtained from each participant in his or her primary language.

### Recruitment

We performed a cross-sectional survey of recently hospitalized CVD patients in Argentina (Buenos Aires, La Plata, Mar del Plata), China (Beijing, Henan Zhoukou City), India (Trivandrum), and Tanzania (Dar es Salaam, Mwanza, Zanzibar). Each country chose three to seven hospitals, including a mix of public and private, urban and rural, hospitals with and without advanced [interventional] treatment facilities to cover a range of hospitalized participants. Hospitalized CVD patients were sampled using a stratified, random-sampling process based on age (<55 years old and ≥55 years old). All decisions related to in-hospital and follow-up medical care were decided by the participant and his/her providing physician. Surveys were developed by experts from the Initiative for Cardiovascular Health Research in Developing Countries to evaluate the individual- and household-level economic impact of hospitalization for a CVD-related event. Questions related to functionality were from adapted from the Short-Form-36.

Standardized surveys (Appendix S1) were translated from English into local languages and culturally adapted by each country site. A pilot study was performed at each site in order to detect implementation difficulties. Surveys were conducted three to fifteen months following hospital discharge. In order to evaluate the economic diversity among the study participants, participants were assigned to one of three income groups based upon the discharge hospital, payment scheme within each hospital, and/or participants' income and/or expenditures. The poorest group represented the poorest 40% (“low”), the middle group represented the middle 40% (“middle”), and the wealthiest group represented the top 20% (“high”) of the national population income distribution.

### Inclusion/Exclusion Criteria

We included participants aged 25–70 years who had been hospitalized for one or more of the following admission diagnoses: acute coronary syndrome (ACS, including unstable angina or myocardial infarction), stroke, acute heart failure, or peripheral vascular intervention (including amputation). First-ever or repeat CVD events were included. Exclusion criteria were any of the following: active malignancy, end-stage renal disease requiring dialysis, solid-organ or hematopoietic transplant, human immunodeficiency virus infection, or severe mental illness.

Survey data were collected in the outpatient clinic or in the household in 2008–2009 by trained personnel to evaluate: 1) demography, 2) medical history, 3) individual and household economic information, 4) expenditures on CVD treatment associated with antecedent CVD event (including indirect costs), and 5) effect of a CVD event on health and productivity. Estimated costs were based upon the total inpatient and follow-up costs up to 15 months and were confirmed by chart review.

### Definitions

A household's health spending was considered *catastrophic* if annual out-of-pocket health expenditures were ≥40% of total, non-food household expenditures, since such spending is likely to result in impoverishment [5], [6]. *Distress financing* was defined as financial activities, such as borrowing money from relatives/friends, taking loans from banks/other lenders, or selling assets (property, e.g.), that were directly related to the patient's most recent hospitalization.

### Statistical Analyses

In order to assess the economic impact of CVD at different income levels, each national group was stratified into income groups. Continuous variables are reported as means ± standard deviation or, if skewed in distribution, as median with interquartile range. Categorical variables are reported as proportions (%). Continuous variables were compared using *t* tests and one-way ANOVA, and categorical variables were compared using the chi-square test. We used univariate and multivariate logistic regression to assess the determinants of catastrophic health spending and distress financing. The multivariate models were constructed using those variables that were significant (p<0.1) in the univariate models and included a dichotomy variable to reflect the type of event, that is 1 for acute coronary syndrome and 0 for stroke. Income and expenditures across all countries were compared using the purchasing power parity conversion to 2007 international dollars (INT$) (7).

## Results

### Demography, Baseline Economic Information, Medical History, and Presentation

Mean participant age ranged from 53 years (Tanzania) to 59 years (China), and the majority of participants were male (Table 1). Median number of years of education completed ranged from seven in Tanzania to ten in India. Median overall monthly individual incomes varied considerably across countries and were lowest in India (INT$259/month) and highest in Argentina (INT$975/month). Significant intra-country variability across income groups was present. A similar pattern was seen for monthly household income. Baseline unemployment ranged from 6% to 24% and was most common in the low-income group. Overall, lack of health insurance was common. Hypertension and tobacco use were among the most common CVD risk factors, and ACS and stroke were the most common admission diagnoses. Median length of stay ranged from 5 to 12 days. Median (interquartile range) time to survey completion following hospital discharge ranged from 174 (83, 264) days in Tanzania to 369 (306, 404) days in China (Table 1).

**Table 1:** Baseline characteristics of survey participants.

	ArgentinaN = 367	ChinaN = 290	IndiaN = 500	TanzaniaN = 498
**DEMOGRAPHICS**				
Mean age, years (SD)	56.6 (8.5)	59.3 (8.1)	56.1 (8.9)	52.9 (11.0)
Male, %	74.1	62.4	79.0	50.2
Married, %	59	94.5	90.0	72.4
Rural, % (National rural prevalence)	3.0 (8)	33.8 (57)	55.0 (70)	42.4 (74)
Median education level, years (IQR)	9 (6, 12)	9 (6, 11)	10 (8,12)	7 (3.5, 10.5)
Median time to survey completion, days (IQR)	251 (148, 354)	369 (306, 404)	240 (150, 330)	174 (83, 264)
Purchasing power parity conversion to INT$1†	1.54 ARS	4.09 RMB	16.54 INR	521 TZS
**INCOME/INSURANCE**				
Median baseline monthly individual income, INT$ (IQR)	975.3*(650, 1,300)*	330.0*(198, 489)*	258.5*(60, 544)*	326.0*(0, 653)*
*Income stratum*				
Low (Lowest 40%)	650.2	73.3	136.1	97.7
Middle (Middle 40%)	1,300.4	220.2	181.4	191.9
High (Highest 20%)	2,600.8*	391.1*	302.4*	767.8*
Median baseline monthly household income, INT$ (IQR)	1,300.4*(813, 1,788)*	611.1*(367, 978)*	453.5*(259, 907)*	768.0*(322, 1,215)*
*Income stratum*				
Low (Lowest 40%)	780.2	122.2	211.6	479.9
Middle (Middle 40%)	1755.5	366.7	302.4	767.8
High (Highest 20%)	3901.2*	855.5*	665.2*	1,919.4*
Dependents <18 years old	36.8	0	46.1	71.7
Dependents >60 years old	42.9	25.0	52.3	48.9
Other individuals in household earning income	96.3	67.0	90.1	97.7
Unemployment, %	16.4	5.9	23.7	6.2
*Income stratum*				
Low (Lowest 40%)	25.5	13.6	42.4	5.6
Middle (Middle 40%)	13.0	7.7	29.3	8.2
High (Highest 20%)	7.5	3.0*	18.4	2.1
Social/private health insurance, %	52.9	80.0	16.5	14.1
**COMORBIDITIES**				
Hypertension, %	56.1	54.5	70.0	88.7
Current/prior tobacco use, %	57.6	10.7	41.0	15.2
Diabetes mellitus, %	19.2	16.6	43.4	16.1
COPD, %	2.7	1.4	3.2	4.4
**HOSPITAL PRESENTATION**				
Acute coronary syndrome, %	66.8	45.9	68.0	1.8
Acute heart failure, %	12.5	0	0	37.1
Peripheral vascular disease, %	1.9	0	0	0.1
Stroke, %	20.0	54.1	32.0	60.4
Median days hospitalized, No. (IQR)	7 (1.5, 12.5)	12 (8, 19)	6 (4, 8)	5 (0, 7)

Mean, median (IQR), and proportions are shown. Differences across income groups are considered statistically significant if p<0.05 (*). Hospital presentation >100% due to multiple causes of hospitalization in Argentina.

†Source: World Bank, available at http://web.worldbank.org/WBSITE/EXTERNAL/DATASTATISTICS/0,contentMDK:20535285~menuPK:1192694~pagePK:64133150~piPK:64133175~theSitePK:239419,00.html, Accessed April 2010.

### Expenditures, Microeconomic Effects, and Income Effects of CVD Event

When CVD expenditure data were stratified by income groups for each nation surveyed, median 15-month out-of-pocket CVD health spending ranged from INT$374 (Tanzania, low-income group) to INT$2,917 (India, high-income group), and median annual household expenditures ranged from INT$1,701 (India, low-income group) to INT$24,597 (Argentina, high-income group)(Table 2). Out-of-pocket CVD expenditures were significantly higher in high-income strata in India and Tanzania but significantly lower in the high-income group in China. The proportion of 15-month out-of-pocket CVD expenditures to annual household expenditures ranged considerably from 4% (Argentina, all income groups) to 55% (India, middle-income group).

**Table 2:** Expenditures and income effects of survey participants are presented.

	ArgentinaN = 367			ChinaN = 290			IndiaN = 500			TanzaniaN = 498		
	Low(n = 76)	Middle(n = 202)	High(n = 89)	Low(n = 44)	Middle(n = 78)	High(n = 168)	Low(n = 66)	Middle(n = 99)	High(n = 335)	Low(n = 200)	Middle(n = 198)	High(n = 100)
**EXPENDITURES**												
Total 15-month out-of-pocket CVD expenditures, INT$ (*IQR*)	477.2*(74, 1,560)*	709.4*(222, 1,951)*	946.0*(142, 2,297)*	1,354.0*(783, 1,914)*	1,366.4*(670, 2,072)*	907.3*(450, 2,110)	773.2*(163, 1,384)*	1,593.4*(382, 2,804)*	2,916.8*(1056, 5,902)	374.3*(118, 630)*	662.3*(106, 1,217)*	1,137.2*(84, 2,190)
Inpatient expenditures/Total CVD expenditures, %	35.7	35.9	31.8	77.2	77.5	82.1	73.8	75.5	81.5*	56.7	57.6	60.7
Annual total household expenditures, INT$ (*IQR*)	12,483.7*(7,542, 17,555)*	17,569.6*(12,109, 24,597)*	24,596.9*(15,501, 34,698)	3,733.3 *(2,421, 4,980)*	4,485.5 *(2,140, 6,628)*	6,067.0*(3,569, 11,929)	1,701.0*(1,288, 2,035)*	2,903.8*(2,519, 3,356)*	7,431.3*(5,233, 9,864)	5,137.4*(3,410, 6,864)*	8,032.6 *(5,135, 10,932)*	16,046.1*(11,037, 21,055)
15-month out-of-pocket CVD expenditures as proportion of annual total household expenditures, %	3.8	4.0	3.8	40.1	30.5	15.0*	45.5	54.9	39.3*	7.3	8.2	7.1
**INCOME EFFECTS**												
Any decrease in individual income, %	78.1	62.5	57.3*	45.5	24.4	13.1*	60.6	38.4	25.1*	68.2	62.1	63.0
Any decrease in household income, %	46.1	47.9	67.5	40.9	25.6	14.3*	62.1	40.4	26.3*	71.9	67.5	63.5
Decrease in individual monthly income since hospitalization, INT$ (*IQR*)	260.1*(20, 390)*	552.7*(325, 780)*	1,267.9*(780, 2,406)	73.3*(49, 122)*	122.2*(49, 244)*	244.4*(98, 367)	37.2*(0, 141)*	0*(0, 121)*	0*(0, 26)	76.8*(0, 192)*	76.8*(0, 192)*	96.0*(0, 576)*
Median decrease in household monthly income since hospitalization, INT$ (*IQR*)	260.1*(130, 455)*	650.2*(374, 910)*	1,950.6*(1,300, 2,666)	85.6*(61, 244)*	122.2*(55, 244)*	342.2*(141, 422)	42.3*(0, 92)*	0*(0, 121)*	0*(0, 30)	96.0*(0, 230)*	96.0*(0, 288)*	191.9*(0, 576)*

Median values (25^th^, 75^th^ percentile) or proportions are presented. P-value<0.05 is considered statistically significant (*). Total 15-month out-of-pocket CVD expenditures included all direct and indirect CVD-related costs after insurance reimbursement (where applicable) for inpatient and follow-up care estimated for the preceding 15 months. Costs included inpatient services, doctors' fees, home care, diagnostic tests, medications, rehabilitation, food, and transportation costs. Annual total household expenditures included monthly estimates of food, energy, transportation, rent/mortgage, education, insurance and annual estimates of durable goods, clothing, fuel, health care, transportation, property management, and other costs estimated by respondents.

Participants from all countries and all income strata reported a decrease in individual incomes after a CVD event. Participants from low-income strata were more likely to report a decrease in individual income than those in the high-income stratum in Argentina, China, and India, but not in Tanzania (Table 2).

**Figure 1:**
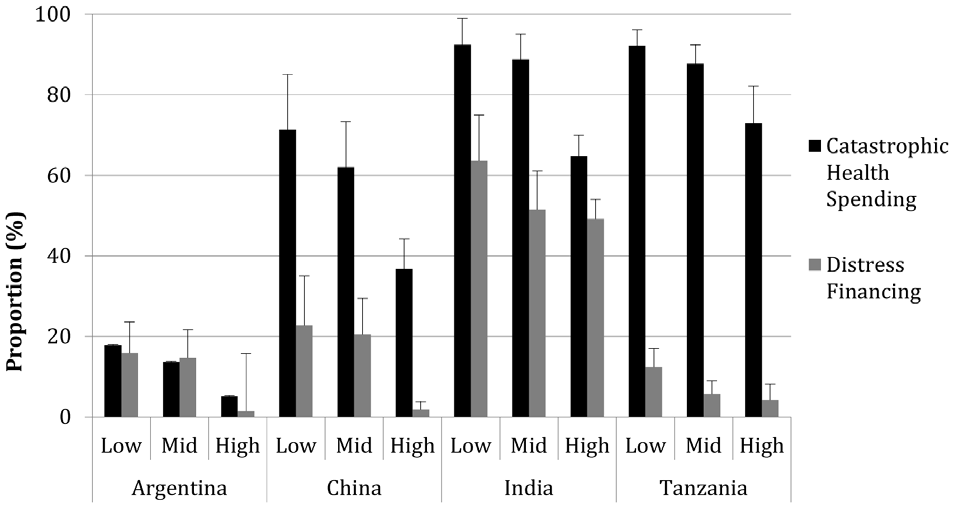
Proportion of survey respondents who experienced catastrophic health spending (out-of-pocket health spending >40% non-food expenditures) and distress financing following CVD-related hospitalization, divided by income strata. Differences across income strata were considered statistically significant (p<0.05) for China (CHS and DF), India (CHS), and Tanzania (CHS and DF).

Catastrophic health spending (CHS) occurred in all countries and income strata, ranging from 5% (Argentina, high-income stratum) to 92% (India and Tanzania, low-income strata). Distress financing ranged from 1% (Argentina, high-income stratum) to 64% (India, low-income stratum) (Figure 1). Borrowing from family, friends, and employers was the most common form of distress financing, but the distribution of distress financing type differed across countries (Appendix S2).

Private/social health insurance was significantly and inversely associated with CHS in all countries by univariate analysis (Tables 3 and 4) and remained significant after controlling for other significant variables in Argentina (OR 4.73 [2.56, 8.76], India (OR 3.93 [2.23, 6.90], and Tanzania (OR 3.68 [1.86, 7.26] with a trend toward association in China (OR 2.05 [0.82, 5.11]). (Table 3) CHS was also associated with the low-income group in China, India, and Tanzania, an association that remained statistically significant in India (OR 6.59 [2.23, 19.45]), after adjustment for other significant variables. Distress financing was predicted by low-income status in Argentina (OR 3.08 [1.12, 8.43]), China (OR 6.67 [1.69, 26.35]), and Tanzania (OR 3.25 [1.08, 9.75]), whereas income status in India was not associated with distress financing (OR 1.30 [0.68, 2.49]). In India, health insurance was the strongest predictor of avoiding distress financing (OR 11.37 [5.18, 24.95]).

**Table 3:** Univariate and multivariate logistic regression models for catastrophic health spending and distress financing by for Argentina and China (see Table 4 for India and Tanzania).

	Catastrophic Health Spending				Distress Financing			
	Univariate analysis	p-value	Multivariate analysis	p-value	Univariate analysis	p-value	Multivariate analysis	p-value
Argentina (n = 367)	OR [95% CI]		OR [95% CI]		OR [95% CI]		OR [95% CI]	
Age Group: <55 vs. >/ = 55 (ref)	1.22 [0.69, 2.17]	0.50	Not in final model	N/A	1.05 [0.68, 1.62]	0.82	Not in final model	N/A
Place of Residence: urban (ref) vs. rural	1.91 [0.24, 15.22]	0.54	Not in final model	N/A	0.41 [0.13, 1.30]	0.13	Not in final model	N/A
Education level: below high school vs. high school or above (ref)	1.01 [0.54, 1.90]	0.98	Not in final model	N/A	0.70 [0.42, 1.17]	0.18	Not in final model	N/A
Employment: Yes (ref) vs. No	1.62 [0.84, 3.10]	0.15	Not in final model	N/A	3.47 [2.06, 5.82]	<0.001	3.45 [2.00, 5.94]	<0.001
Social/private insurance: Yes (ref) vs. No	4.07 [2.33, 7.11]	<0.001	4.73 [2.56, 8.76]	<0.001	1.31 [0.85, 2.04]	0.22	Not in final model	N/A
Highest income group (ref)	*Reference*		*Reference*		*Reference*		*Reference*	
Middle income group	2.91 [0.66, 12.83]	0.16	Not in final model	N/A	2.74 [1.02, 7.36]	0.046	2.60 [0.95, 7.07]	0.06
Lowest income group	3.99 [0.91, 17.59]	0.07	Not in final model	N/A	3.79 [1.41, 10.21]	0.01	3.08 [1.12, 8.43]	0.03
Presentation: ACS (ref) vs. stroke	2.25 [0.97, 5.20]	0.06	Not in final model	N/A	0.82 [0.49, 1.37]	0.454	Not in final model	N/A
**China (n = 290)**								
Age Group: <55 vs. >/ = 55 (ref)	0.50 [0.29, 0.86]	0.01	0.47 [0.26, 0.85]	0.01	1.48 [0.67, 3.28]	0.33	Not in final model	N/A
Place of Residence: urban (ref) vs. rural	4.86 [2.75, 8.58]	<0.001	2.69 [1.31, 5.53]	0.01	12.07 [4.44, 32.81]	<0.001	5.13 [1.53, 17.13]	0.008
Education level: below high school vs. high school or above (ref)	1.57 [0.93, 2.65]	0.09	Not in final model	N/A	2.37 [0.88, 6.43]	0.09	Not in final model	N/A
Employment: Yes (ref) vs. No	0.93 [0.35, 2.49]	0.89	Not in final model	N/A	1.21 [0.26, 5.58]	0.81	Not in final model	N/A
Social/private insurance: Yes (ref) vs. No	5.62 [2.75, 11.50]	<0.001	2.05 [0.82, 5.11]	0.13	7.57 [3.36, 17.02]	<0.001	1.36 [0.49, 3.81]	0.56
Highest income group (ref)	*Reference*		*Reference*		*Reference*		*Reference*	
Middle income group	4.31 [2.05, 9.07]	<0.001	2.40 [1.03, 5.56]	0.04	16.08 [4.20, 61.53]	<0.001	7.23 [1.65, 31.71]	0.009
Lowest income group	2.81 [1.58, 5.01]	<0.001	1.62 [0.84, 3.11]	0.13	14.11 [3.97, 50.10]	<0.001	6.67 [1.69, 26.35]	0.007
Presentation: ACS (ref) vs. stroke	1.00 [0.62, 1.61]	0.99	Not in final model	N/A	1.06 [0.49, 2.28]	0.89	Not in final model	N/A

The multivariate models were constructed using variables that were significant (p<0.1) in the univariate models and included a dichotomy variable to reflect the type of event, that is 1 for acute coronary syndrome and 0 for stroke.

**Table 4:** Univariate and multivariate logistic regression models for catastrophic health spending and distress financing by for India and Tanzania (see Table 3 for Argentina and China).

	Catastrophic Health Spending				Distress Financing			
	Univariate analysis	p-value	Multivariate analysis	p-value	Univariate analysis	p-value	Multivariate analysis	p-value
India (n = 500)	OR [95% CI]		OR [95% CI]		OR [95% CI]		OR [95% CI]	
Age Group: <55 vs. >/ = 55 (ref)	1.68 [1.13, 2.51]	0.01	1.66 [1.06, 2.61]	0.03	0.69 [0.49, 0.99]	0.04	0.57 [0.38, 0.87]	0.009
Place of Residence: urban (ref) vs. rural	1.93 [1.29, 2.90]	<0.001	1.28 [0.82, 2.00]	0.28	2.11 [1.47, 3.03]	<0.001	1.93 [1.27, 2.93]	0.002
Education level: below high school vs. high school or above (ref)	1.83 [1.09, 3.09]	0.02	1.00 [0.54, 1.86]	1.00	2.10 [1.36, 3.25]	<0.01	2.27 [1.34, 3.86]	0.002
Employment: Yes (ref) vs. No	1.83 [1.10, 3.05]	0.02	0.90 [0.50, 1.60]	0.71	1.45 [0.95, 2.20]	0.08	Not in final model	N/A
Social/private insurance: Yes (ref) vs. No	4.42 [2.63, 7.41]	<0.001	3.93 [2.23, 6.90]	<0.001	11.19 [5.24, 23.92]	<0.001	11.37 [5.18, 24.95]	<0.001
Highest income group (ref)	*Reference*		*Reference*		*Reference*		*Reference*	
Middle income group	4.32 [2.22, 8.41]	<0.001	3.66 [1.83, 7.30]	<0.01	1.10 [0.70, 1.73]	0.67	0.96 [0.58, 1.61]	0.885
Lowest income group	6.67 [2.61, 17.04]	<0.001	6.59 [2.23, 19.45]	<0.001	1.81 [1.05, 3.13]	0.03	1.30 [0.68, 2.49]	0.429
Presentation: ACS (ref) vs. stroke	0.96 [0.63, 1.46]	0.84	0.60 [0.37, 0.97]	0.04	0.50 [0.34, 0.74]	<0.001	0.32 [0.21, 0.51]	<0.001
**Tanzania (n = 498)**								
Age Group: <55 vs. >/ = 55 (ref)	1.41 [0.84, 2.36]	0.20	Not in final model	N/A	0.90 [0.47, 1.73]	0.76	Not in final model	N/A
Place of Residence: urban (ref) vs. rural	1.99 [1.13, 3.49]	0.01	2.00 [1.07, 3.73]	0.03	0.83 [0.43, 1.63]	0.60	Not in final model	N/A
Education level: below high school vs. high school or above (ref)	2.35 [1.37, 4.03]	<0.001	1.47 [0.79, 2.74]	0.22	1.24 [0.53, 2.87]	0.62	Not in final model	N/A
Employment: Yes (ref) vs. No	1.05 [0.94, 1.17]	0.37	Not in final model	N/A	1.00 [0.87, 1.15]	0.99	Not in final model	N/A
Social/private insurance: Yes (ref) vs. No	4.71 [2.59, 8.59]	<0.001	3.68 [1.86, 7.26]	<0.001	6.91 [0.93, 51.10]	0.06	Not in final model	N/A
Highest income group (ref)	*Reference*		*Reference*		*Reference*		*Reference*	
Middle income group	2.65 [1.41, 5.00]	<0.001	2.14 [0.98, 4.64]	0.05	1.38 [0.43, 4.47]	0.59	Not in final model	N/A
Lowest income group	4.37 [2.13, 8.98]	<0.001	2.34 [0.68, 8.05]	0.17	3.25 [1.08, 9.75]	0.03	3.25 [1.08, 9.75]	0.03
Presentation: ACS (ref) vs. stroke	1.72 [0.35, 85.69]	0.47	Not in final model	N/A	0.91 [0.11, 7.50]	0.93	Not in final model	N/A

The multivariate models were constructed using variables that were significant (p<0.1) in the univariate models.

#### Argentina

In Argentina, 15-month out-of-pocket CVD expenditures were similar across all income strata with a trend toward increased costs in the high-income group. Private/social health insurance coverage was moderate (52%) across all respondents. Access to ambulatory and in-hospital care is fully granted for all Argentineans, regardless of their insurance status or hospital where they receive care. In this context, CHS was relatively low across all groups but was significantly associated with lack of private or social health insurance (OR 4.72 [2.56, 8.76] Table 3). The proportion of distress financing was similar across all income groups and was significantly associated low-income status (OR 3.08 [1.12, 8.43]). Nevertheless, more than half of all participants from Argentina reported that they had experienced a decrease in individual and household income following their CVD-related hospitalization (Table 2).

#### China

Overall out-of-pocket CVD expenditures were higher among participants in the low-income stratum; the ratio of out-of-pocket CVD spending to total household expenditures was also higher. CHS was highest in the low-income stratum (71%) and had a significant negative association with age <55 years (OR 0.47 [0.26, 0.85]) and significant positive association with rural status (OR 2.69 [1.31, 5.53]) (Table 3). A similar distribution was seen for distress financing, which was significantly associated with rural status (OR 5.13 [1.53, 17.13] and low-income group (OR 6.67 [1.69, 26.35]). The subsequent proportion of participants who experienced any decrease in income was highest in the low-income group (53%), though the absolute decrease was highest in the high-income group (Table 2).

#### India

India had the highest 15-month out-of-pocket CVD expenditures among the comparator countries. Insurance coverage was uncommon in Indian respondents (16%), and overall CHS was common and regressive. CHS was associated with age <55 years (OR 1.66 [1.06, 2.61]), lack of private/social health insurance (OR 3.93 [2.23, 19.45]), and stroke (OR 0.60 [0.37, 0.97]) (Table 4). A similar pattern was seen for distress financing, which was again higher than other comparator countries. Rural status (OR 1.93 [1.27, 2.93]), less than secondary school education (OR 2.27 [1.34, 3.86], absence of private/social health insurance (OR 11.37 [5.18, 24.95]), and stroke (OR 0.32 [0.21, 0.51]) were significantly associated with distress financing. The proportion of participants who experienced any decrease in income was highest in the low-income stratum (43%), but the absolute decrease was also highest in the low-income group (Table 2).

#### United Republic of Tanzania

In Tanzania, increased 15-month out-of-pocket CVD expenditures were associated with the high-income respondents, as were annual household costs. Insurance coverage was low throughout Tanzania (14%). As such, CHS was high in all income groups and was associated with rural status (OR 2.00 [1.07, 3.73]) and lack of private/social health insurance (OR 3.68 [1.86, 7.26] Table 4). Distress financing was uncommon, however, and ranged from 4% to 12% and was only associated with low-income group (OR 3.25 [1.08, 9.75]). Nevertheless, two-thirds of respondents reported a decrease in income following their CVD-related hospitalization. The proportion of respondents experiencing any decrease in income was similar across income groups (Table 2).

### Functional Capacity and Productivity

The health effects, functional effects, and household effects of CVD-related hospitalization demonstrate the potential indirect economic impact of CVD-related hospitalization (Table 5). Most respondents experienced functional limitations in moderate and vigorous activities with subsequent emotional distress following their hospitalization. Individual decreases in work time and work activities were common coping mechanisms, whereas families responded by a net increase (Argentina, China, India) or net decrease (Tanzania) in work time to compensate for their family member's disability.

**Table 5:** Functional health effects, productivity effects, and household effects of CVD-related hospitalization among respondents from Argentina, China, India, and Tanzania.

	ArgentinaN = 367			ChinaN = 290			IndiaN = 500			TanzaniaN = 498		
	Low (n = 76)	Middle (n = 202)	High (n = 89)	Low (n = 44)	Middle (n = 78)	High (n = 168)	Low (n = 66)	Middle (n = 99)	High (n = 335)	Low (n = 200)	Middle (n = 198)	High (n = 100)
**FUNCTIONAL HEALTH EFFECTS**												
Reporting decrease in self-rated health, %	47.4	45.0	52.8	61.4	60.3	57.7	60.6	64.6	58.7	94.0	95.9	98.0
Decreased moderate activity ability, %	86.8	86.1	86.5	47.7	61.5	54.2	42.4	49.5	44.0	79.4	88.2	86.5*
Decreased vigorous activity ability, %	90.8	86.6	88.8	81.8	83.3	73.2	66.7	63.6	66.2	92.3	95.3	97.2
Experiencing emotional problems, %	72.4	59.4	57.3	40.9	50.0	50.4	9.1	20.2	32.5	61.0	73.2	80.0
Unable to take medications due to cost, %	13.3	7.3	10.6	43.8	29.6	7.1*	6.1	10.1	8.1	94.4	99.5	99.0*
**PRODUCTIVITY EFFECTS**												
Decreased work time, %	77.6	75.7	70.4	90.9	87.2	70.2*	90.9	87.9	81.7	98.9	98.5	100.0
Limited work activities, %	86.8	78.2	74.7	86.4	92.3	85.7	90.9	90.9	85.9	98.9	98.5	100.0
Feeling limited, %	86.7	65.8	57.3	90.9	92.3	88.7	89.4	90.8	85.7	98.5	98.5	99.0
**HOUSEHOLD EFFECTS**												
Decreased work time (or stopping work) by family members, %	11.8	9.9	6.7	20.5	17.9	10.1	4.6	4.3	2.4	18.9	21.4	24.7
Increased work time (or starting work) by family members, %	17.1	20.8	22.5	34.1	24.4	7.1*	13.8	8.2	5.7	16.3	14.9	11.3

Differences across income strata were considered statistically significant if p<0.05 (*).

#### Argentina

Approximately 50% of participants across all income strata reported a decrease in their health with nearly all reporting a decrease in their ability to participate in moderate (86%) or vigorous (88%) activities. The proportion of individuals experiencing emotional problems following their CVD-related hospitalization was also high (61%). Cost was the primary reason for not taking one's medications in 7–13% of respondents.

Most respondents reported a decrease in work time (73%) and reported limiting their work activities (78%) following their CVD-related hospitalization. The low-income stratum was more likely to report feeling limited overall (87%), but this response was common for the entire group (67%). Family members were more likely to increase their work time (or start new work) rather than report a decrease in their work time (or stop work)(Table 5).

#### China

The majority of respondents reported a decrease in their self-rated health (62%), while half (51%) of participants were less able to perform moderate physical activities and three-fourths (76%) of participants were less able to perform vigorous physical activities. Approximately one-fourth of the respondents did not take their medications due to cost (Table 5).

Most respondents decreased their work time (81%) and limited their work activities (87%) following their CVD-related hospitalization. The overall proportion of family members who increased their work time (14%) was similar to the proportion that decreased their work time (15%), but the increase in work time by family members was most common in the low-income stratum (Table 5).

#### India

Almost two-thirds of respondents reported a decrease in their self-rated health (60%). Less than half of participants reported a worsening of their ability to perform moderate activities (45%), while two-thirds reported a worsening of their ability to perform vigorous activities, both of which trended lower than the other comparator countries. The proportion of individuals reporting emotional problems following their CVD-related hospitalization was also lower than the comparator countries (27%) but highest in the high-income strata (33%). Approximately one in ten respondents across all income strata did not take their medications due to cost (Table 5).

More than three-fourths of respondents decreased their work time, limited their work activities, and felt limited overall. A slightly greater proportion of family members increased their work time rather than decrease their work time following the respondent's CVD-related hospitalization, and this trend was most marked in the low-income stratum (Table 5).

#### United Republic of Tanzania

Nearly all respondents across all income strata reported a decrease in their self-rated health (96%) with a corresponding worsening in their ability to participate in moderate (84%) and vigorous (96%) activities. The proportion of individuals reporting emotional problems following their CVD-related hospitalization was high overall (70%) and highest in the high-income strata. Approximately one-third of the respondents did not take their medications as prescribed, which was largely due to the highs costs (76%) (Table 5).

Nearly all respondents decreased their work time, limited their work activities, and felt limited overall (99%) following their CVD-related hospitalization. A greater proportion of family members decreased their work time rather than increased their work time to care for the patient (Table 5).

## Discussion

### Health Care Spending

We evaluated the individual- and household-level impact of CVD-related hospitalization across four LMIC and found that 15-month out-of-pocket CVD expenditures varied considerably across countries and across income groups within countries (INT$374 [Tanzania, low-income] to INT$2,917 [India, high-income]). By comparison, in-hospital out-of-pocket expenditures for CVD in the United States were estimated to be INT$1,229 in 2006 [7].

In two of the four countries studied, CVD costs are regressive: poorer respondents pay a higher proportion of income on health care following a CVD hospitalization, as previously demonstrated for other conditions in other countries [8], [9]. The examples of Argentina and Tanzania in our survey shows that regressive CVD costs are not universal in LMIC [10] and suggests there is a wide range of country-specific, economic and/or health system development within the LMIC category.

### Financing Mechanisms and Impoverishing Effects of CVD

Catastrophic health spending was common in China, India, and Tanzania and most strongly impacted the poorest CVD patients and their families. Our results are markedly higher than mean levels of CHS previously reported among community dwellers in 89 countries (2.3%) [4], [11]. Global estimates for CHS range from almost zero percent in the United Kingdom, Czech Republic, and Slovakia to >10% percent in Vietnam and Brazil [11]. However, since our study focused on recently hospitalized patients, our figures would be expected to be higher, though perhaps not to this degree. The high proportion of CHS may be underestimated because of individuals who avoid medical treatment due to high, perceived costs, yet might have financial insecurity if they did. On the other hand, distress financing—risky financial activities such as borrowing loans and selling assets—was present in >40% of participants only in India. Participants from Argentina, China, and Tanzania most commonly borrowed money from family, friends, and employers to cover their health care costs, whereas more than half of participants who experienced distress financing in India borrowed from banks/moneylenders or sold assets.

A 2009 survey evaluating the microeconomic impact of stroke across 62 hospitals in China found that 71% of post-stroke patients reported CHS. While the authors used a different definition of CHS (≥30% of annual income), the findings are similar overall to our results (the range of CHS in our sample from China across income groups was 37–71%) [13]. However, in our sample, CHS was not associated with hospitalization due to stroke compared with acute coronary syndromes, which may be due to higher costs of acute coronary syndromes or a lack of power to detect such a difference. This comparison across Argentina, China, India, and Tanzania provides a wider scope of the individual- and household-level economic impact of CVD-related hospitalizations.

Along with high CVD-related costs, many participants reported decreased income, poorer perceived health (including emotional problems), lower functional and productivity capacity, and variable household effects, all of which likely exacerbated their financial instability. These findings, coupled with the relatively low proportion of any form of insurance, likely contributed to the high proportion of CHS. Some argue that CHS overestimates the impoverishing effects of health care costs since families are able to “smooth consumption” by drawing upon savings, assets, credit and loans from friends and relatives [12]. We tried to account for such activities through questions about distress financing, which may offset CHS but contributes to chronic impoverishment [12].

Lack of private/social health insurance was significantly associated with an increased risk of CHS in 3 of 4 countries studied and a trend toward association in China. Insurance alone, however, does not protect fully against CHS, as evident in our study with the high proportion of CHS in China despite a relatively higher proportion of insurance coverage. This discrepancy may be due, in part, to the proportion of reimbursement by the insurer, which was lowest for both hospital and outpatient charges in the low-income group and rural respondents in China (data not shown). On the other hand, low-income respondents were more likely to experience distress financing in 3 of 4 countries, suggesting that poorer participants may be less likely to have financial reserves to bear the costs of a CVD-related hospitalization. The availability of health services requiring payment, national inequality in health spending, and the capacity of individuals and households to pay (non-subsistence spending) are other key determinants associated with CHS [11].

### Solutions to Avoid Catastrophic Health Spending and Distress Financing

The primary means to avoid distress financing typically includes prepayment either through tax financing, social health insurance programs, or private health insurance programs, the latter two which have been shown to reduce CHS incidence in Mexico [13]. On the other hand, social health insurance programs have been shown to increase per capita health spending by 3–4% without improved outcomes or even at the cost of 5–6% potential life years lost in one report [14], [15]. Other studies have demonstrated an increase in CHS with a decrease in the depth of insurance, including evaluations of China's urban and rural insurance schemes, which promote more complex, expensive care that is not wholly covered [16], [17]. Another mechanism to decrease CHS and distress financing is to reduce the supply side of health care through restrictions in spending opportunities (treatment protocols, essential drug lists, restriction of unnecessary interventions, e.g.), which has been shown to be more successful at reducing CHS in China than expansion of insurance coverage alone [18].

### Limitations

Our study has inherent limitations. First, our survey sample was hospital-based, and did not sample patients who avoided seeking care for a CVD event nor those who did not survive a CVD event during the follow-up period: this may bias the results by describing only the most severe events, or by missing patients who avoided seeking medical care or those who did not survive a CVD event. Second, participants were asked to report sensitive income and expenditure information up to 15 months after hospitalization, which may be susceptible to reporting bias. Third, our survey captured participants from selected hospitals from each country, which should not be considered generalizable to other hospitals. We did, however, attempt to capture a large number of respondents to effectively evaluate the economic effects of CVD-related hospitalizations in these selected LMIC, though country sample sizes per country were not proportionate to overall country population sizes. Fourth, our sample size may have been too small to detect differences in the distribution and determinants of CHS and DF. Fifth, many patients could not afford costly treatments like medications, percutaneous coronary intervention or coronary artery bypass graft surgery nor diagnostics and follow-up care and subsequently did not undergo them, which may underestimate expenditures in an evidence-based treatment setting. Sixth, we did not exhaust all the potentially relevant microeconomic consequences of CVD hospitalization, such as savings or labor supply.

### Conclusions

Patients in Argentina, China, India, and Tanzania bear a significant burden of out-of-pocket payments, as defined by CHS and DF, following CVD hospitalization, though substantial variations exist across and within countries. Lack of insurance appears to be a major, remediable source of the financial burden of CVD in these countries. As CVD prevalence increases in LMIC, the household economic impact of CVD may worsen without the development of alternative health spending models that enhance patients' capacity to pay or without more active policies to prevent or at least postpone the onset of CVD in LMIC.

#### Steering Committee

Dorairaj Prabhakaran, Stephen Leeder, K. Srinath Reddy, Krishna D. Rao, Andres Pichon-Riviere, Dong Zhao, S. Harikrishnan, Kaushik Ramaiya, Thomas A. Gaziano, Lorenzo Rocco, Marc Suhrcke, Andrew E. Moran.

#### Coordinating Centre

V.S. Ajay, Krishna D. Rao, Poornima Prabhakaran, Shifalika Goenka, Mark D. Huffman, K. Srinath Reddy, Dorairaj Prabhakaran.

#### Centre Staff

Juan Calcagno, Joaquín Caporale, Shaoli Niu, Li Yan, Jing Liu, K.R. Thankappan, Meena Daivadanam, Jan van Esch, Julie S., Diana S., Renju T.S.

## Supporting Information

Appendix S1 English language example of survey.
(TIF)

Appendix S2 Patterns of distress financing across Argentina, China, India, and Tanzania following CVD-related hospitalization. (Gray = sold land or other assets; black = borrowed money from bank or moneylenders; red = borrowed money from friends, family, and employer).
(DOC)
