# Supplementary figures and images for: A Cross-Sectional Study of the Microeconomic Impact of Cardiovascular Disease Hospitalization in Four Low- and Middle-Income Countries
Source: PLoS One. 2011 Jun 14;6(6):e20821. doi: 10.1371/journal.pone.0020821 (PMC3114849; doi:10.1371/journal.pone.0020821)

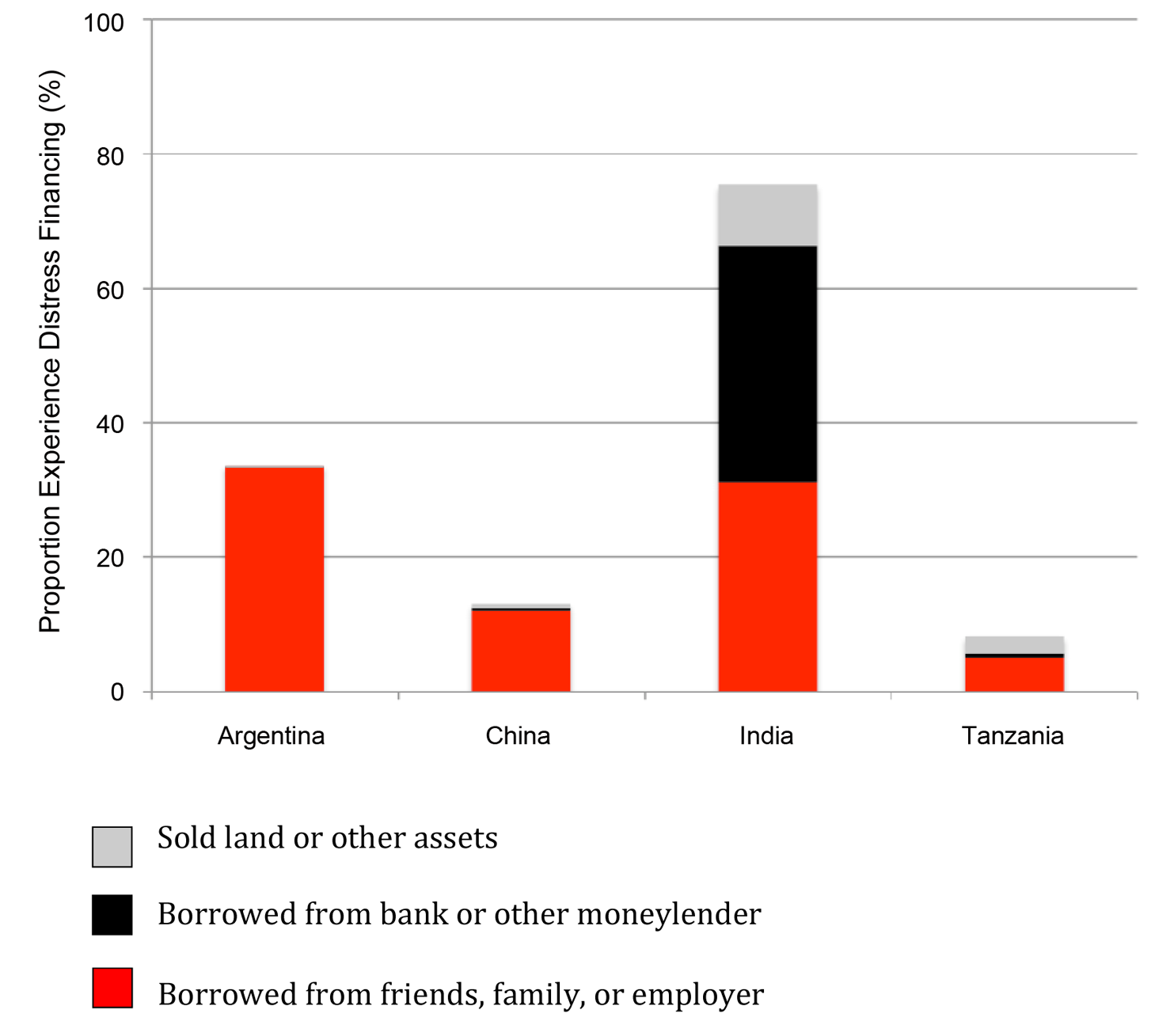

Supplement: Appendix S1 — English language example of survey. (TIF) [file pone.0020821.s001.tif]

**Appendix 2. English language example of survey.**


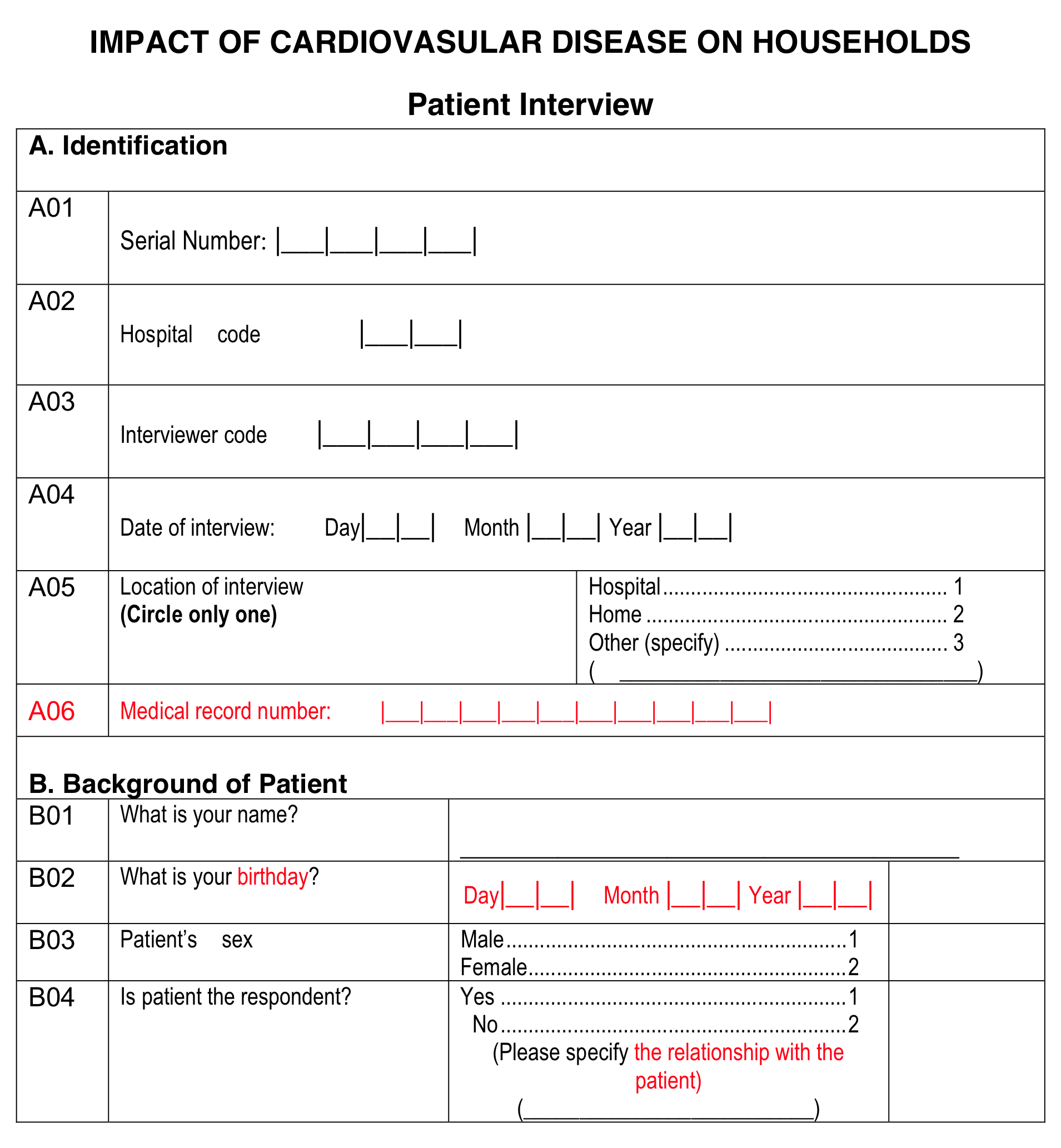


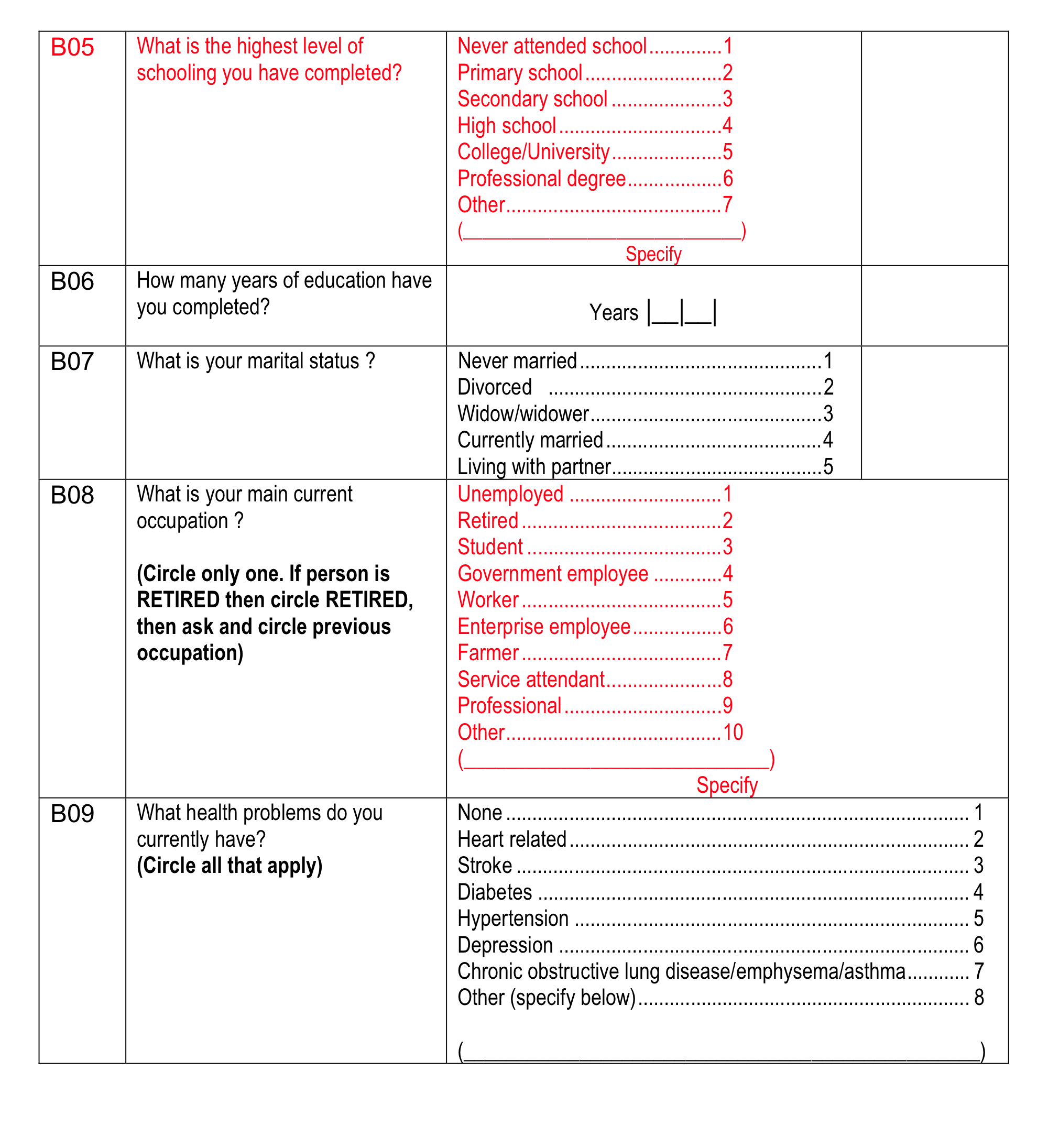


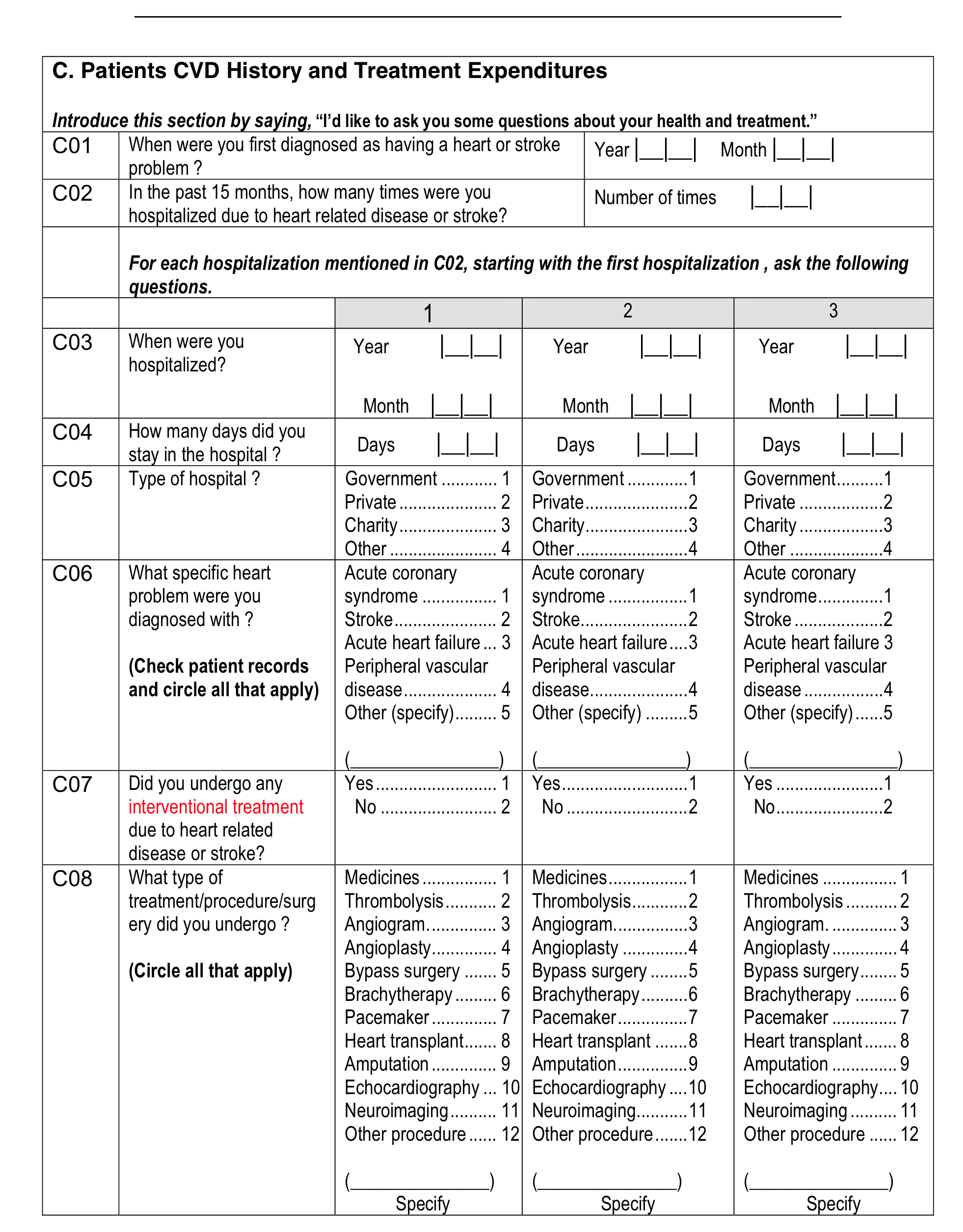


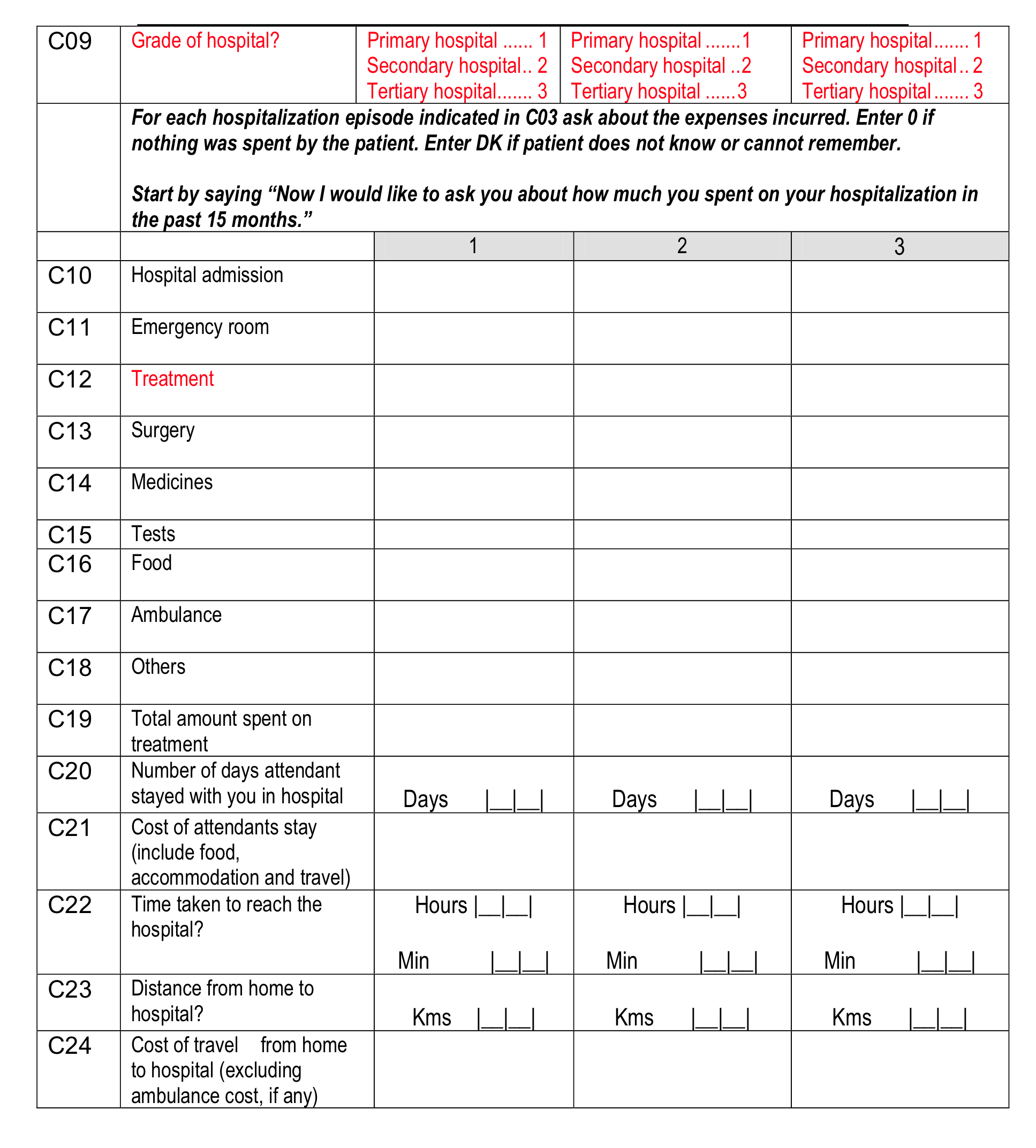


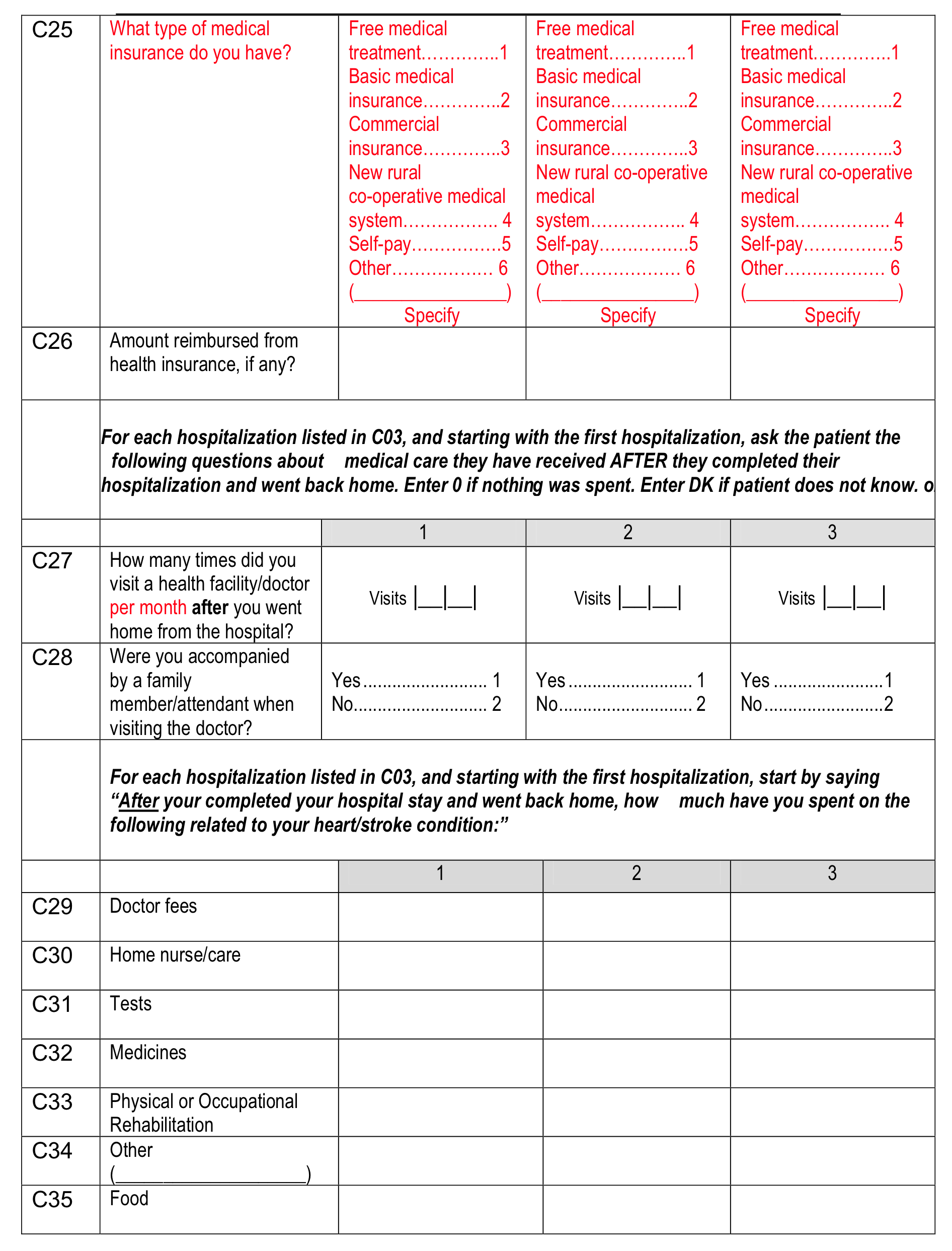


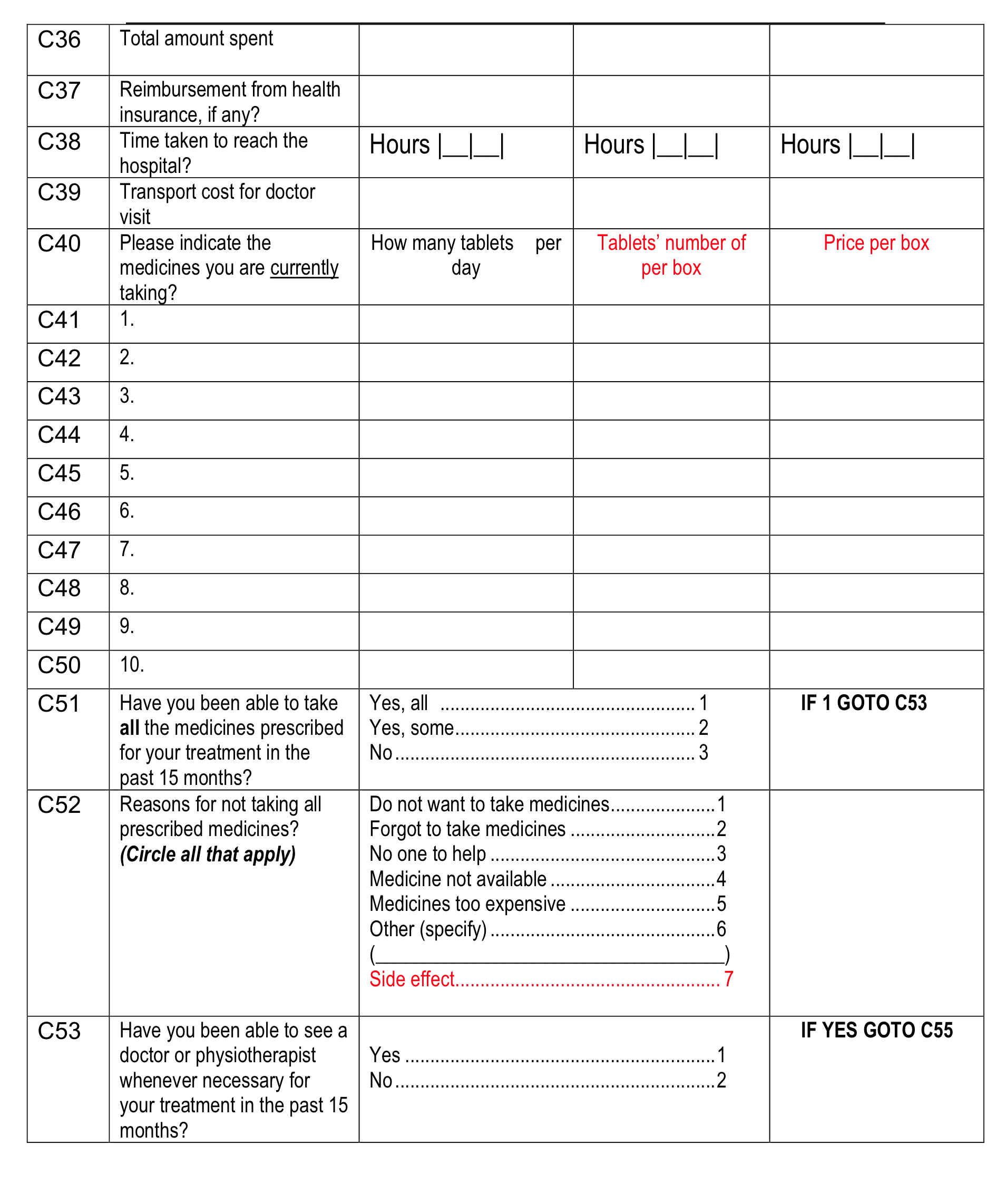


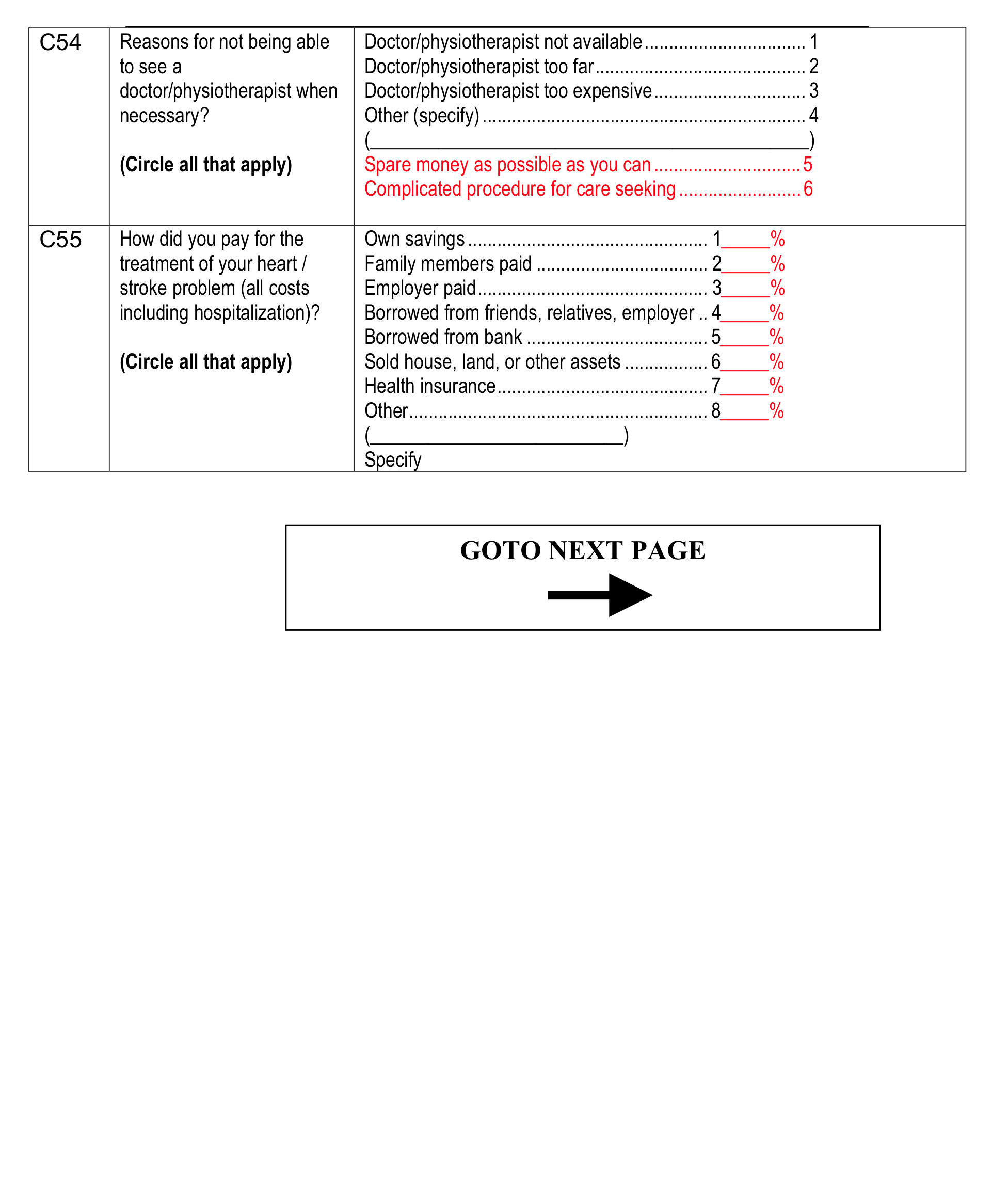


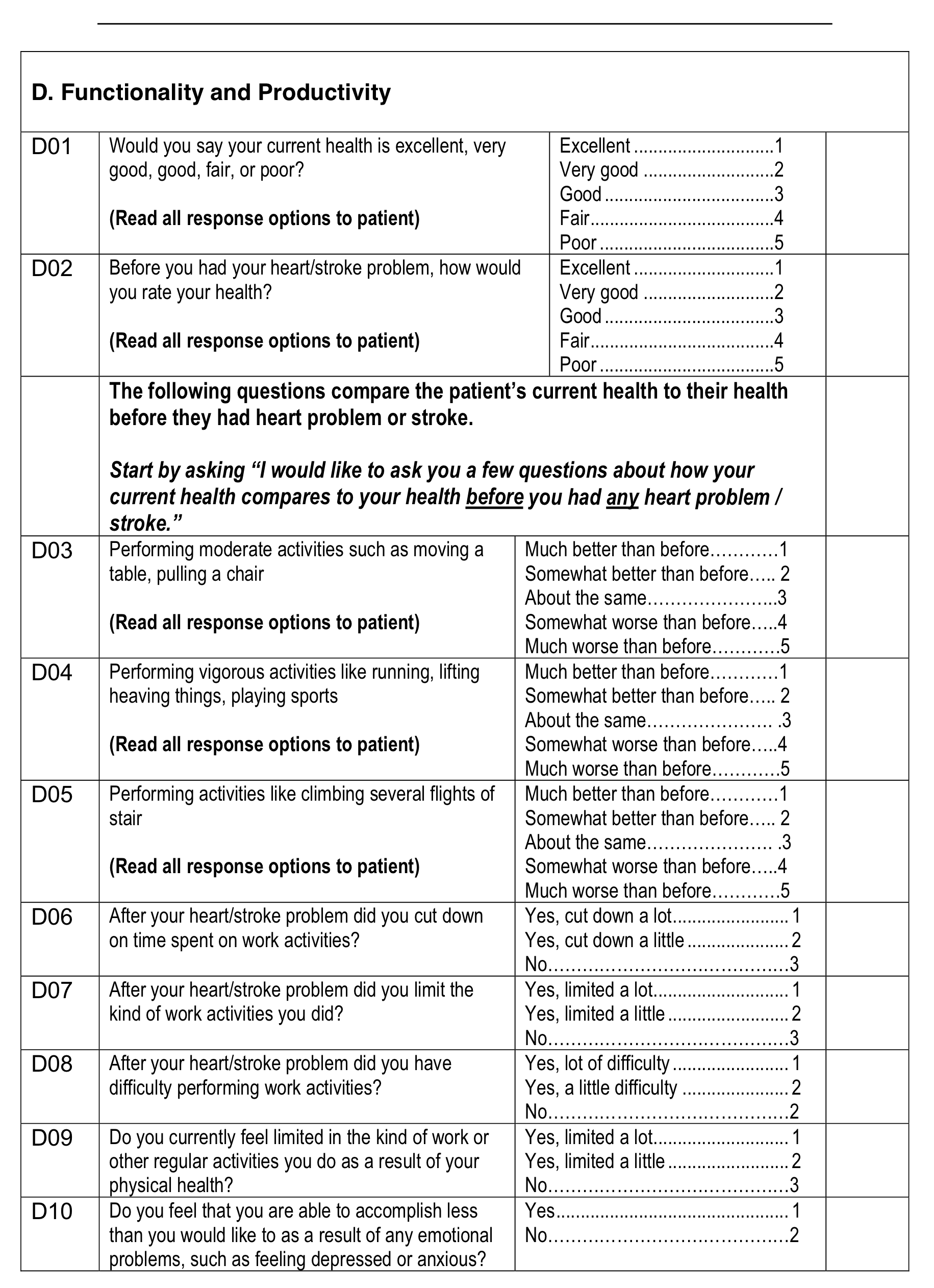


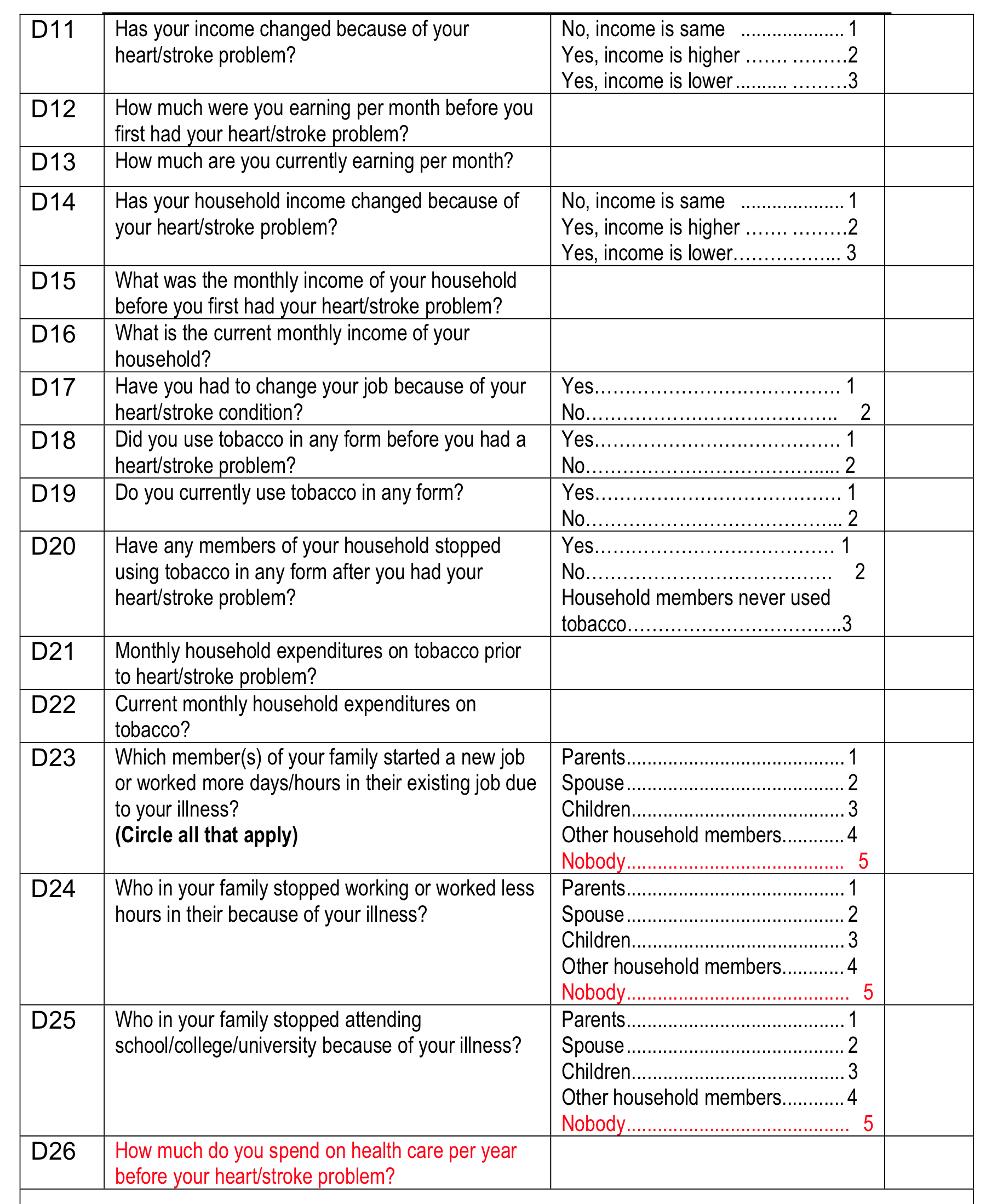


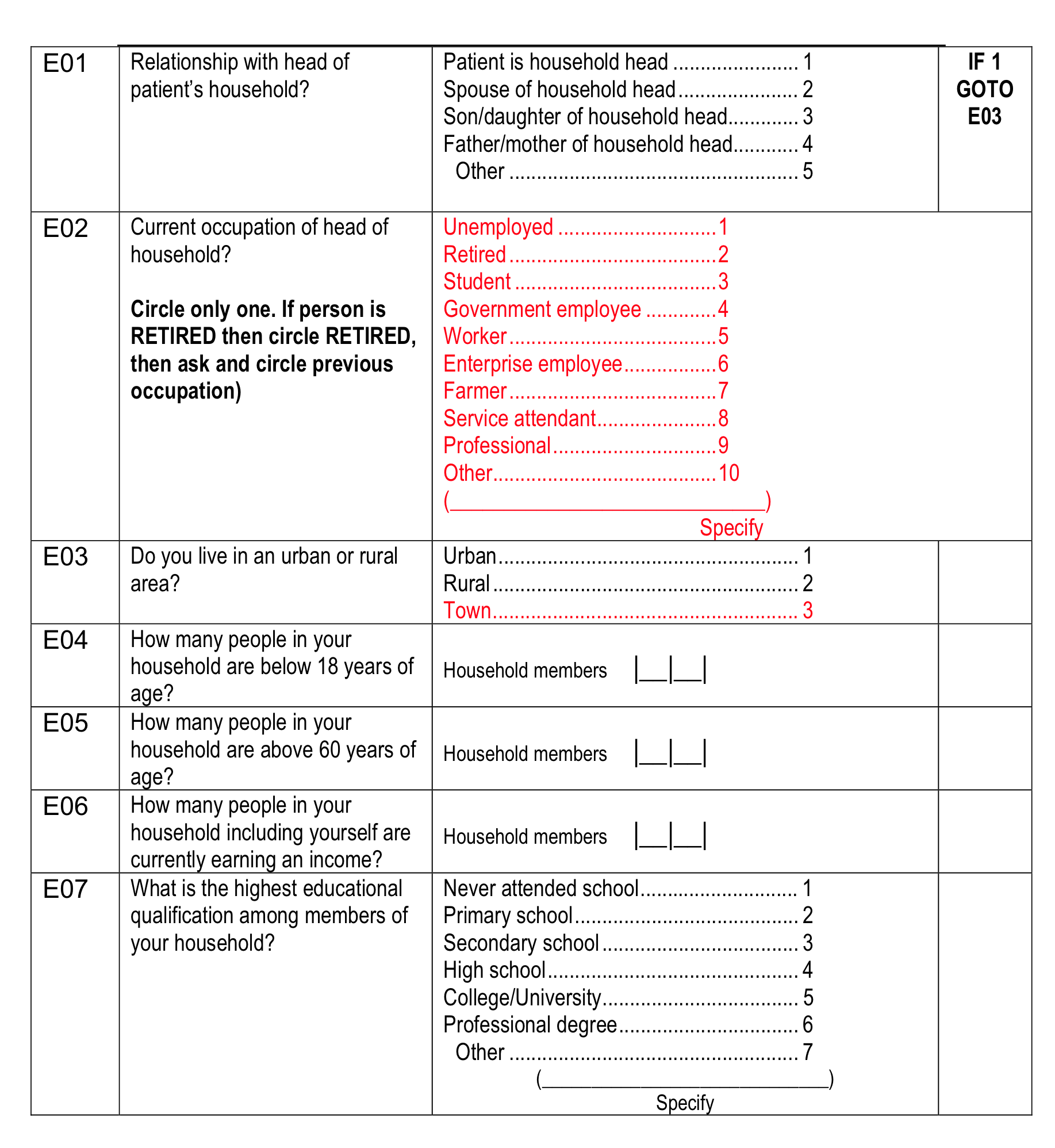


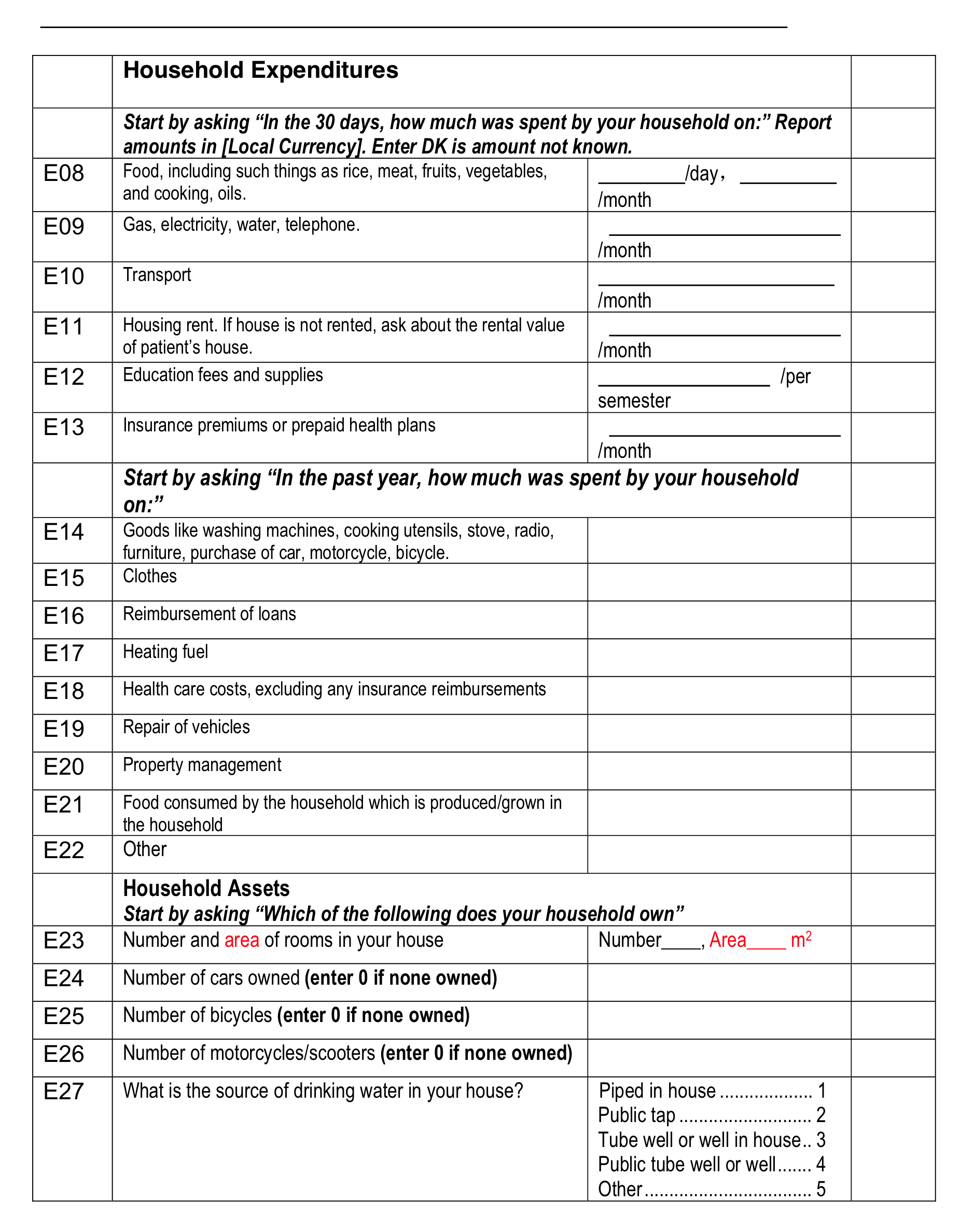


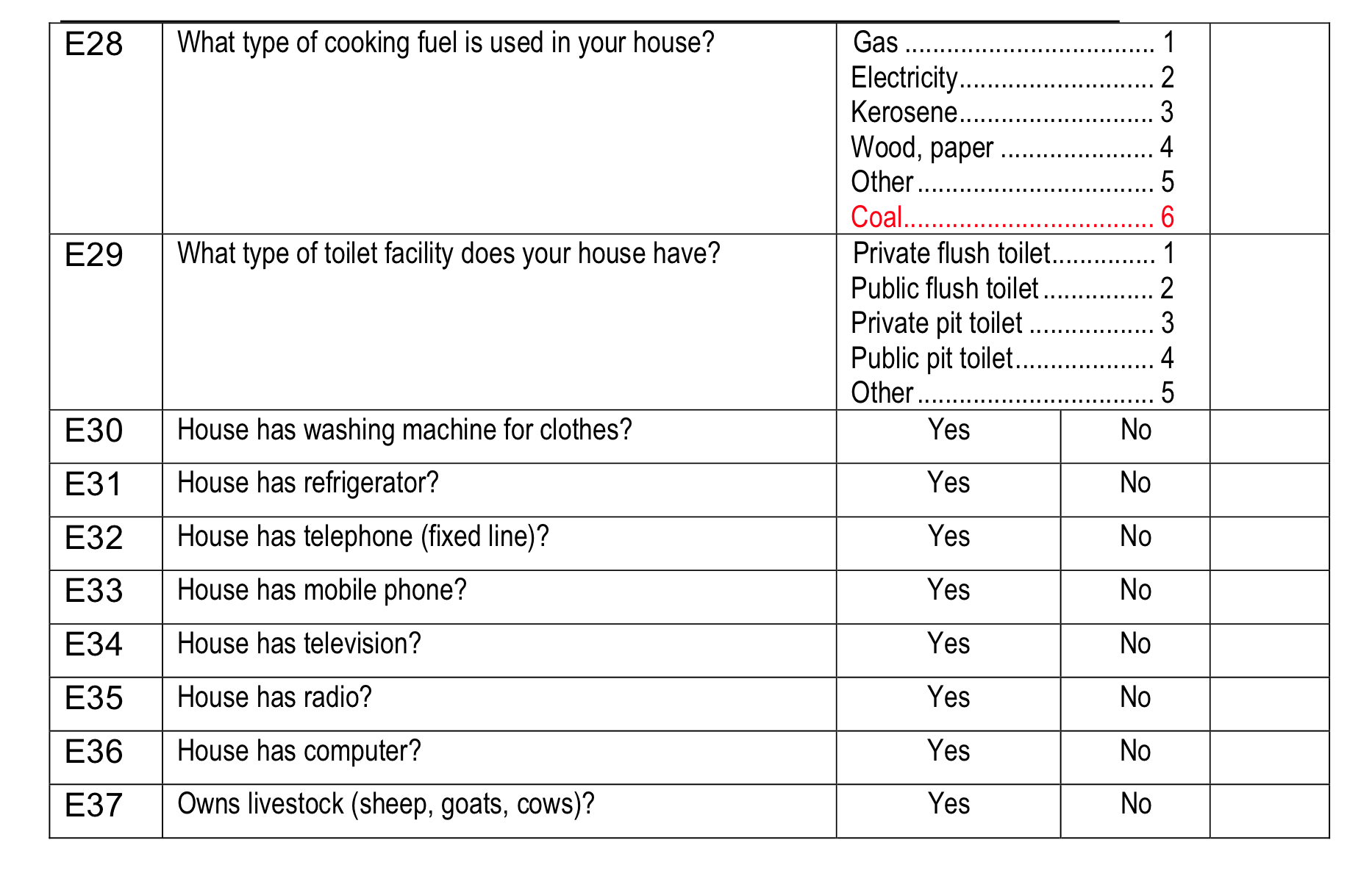

Supplement: Appendix S2 — Patterns of distress financing across Argentina, China, India, and Tanzania following CVD-related hospitalization. (Gray = sold land or other assets; black = borrowed money from bank or moneylenders; red = borrowed money from friends, family, and employer). (DOC) [file pone.0020821.s002.doc]
